# Moving beyond hydroxychloroquine: the novel lysosomal autophagy inhibitor ROC-325 shows significant potential in preclinical studies

**DOI:** 10.1186/s40880-019-0418-0

**Published:** 2019-11-09

**Authors:** Trace M. Jones, Claudia Espitia, Wei Wang, Steffan T. Nawrocki, Jennifer S. Carew

**Affiliations:** 1 University of Arizona Cancer Center, Tucson, AZ 85724 USA; 2 Division of Translational and Regenerative Medicine, Department of Medicine, University of Arizona, Tucson, AZ 85721 USA; 3 Department of Pharmacology and Toxicology, University of Arizona, Tucson, AZ 85721 USA; 4 Arizona Center for Drug Discovery, Tucson, AZ 85721 USA

Autophagy is a lysosomal-mediated degradation process that controls the turnover of organelles and long-lived proteins. Outside of its role in maintaining cellular homeostasis, autophagy is frequently stimulated in response to stress conditions such as those imposed by chemotherapy, nutrient deprivation, and microenvironmental disruption. In this capacity, autophagy serves as a mechanism to generate alternative sources of metabolic fuel that promote survival when preferred energy generating pathways are impaired [1]. Over the past decade, stress-induced autophagy has emerged as an important driver of malignant progression and anticancer drug resistance. Virtually all classes of clinically relevant anticancer agents, as well as radiotherapy, have been demonstrated to stimulate autophagy in preclinical studies [2]. In the overwhelming majority of cases, genetically or pharmacologically impairing autophagy has been shown to yield therapeutic benefit in a manner that significantly improves the efficacy of the relevant agents or modalities [3].

The impressive boost in efficacy that was achieved for such a wide range of anticancer therapeutics in preclinical studies prompted a very obvious question: how can we translate this into patient benefit? This was initially thought to be a minor hurdle to overcome. A very old drug (hydroxychloroquine (HCQ)) that had been used for decades in the management of malaria, lupus, and rheumatoid arthritis was approved by the US Food and Drug Administration (FDA) and immediately available for use in early phase combination therapy cancer clinical trials. Although lacking in significant single-agent anticancer activity, HCQ had been shown to augment the efficacy of many anticancer regimens through its off-target ability to disrupt lysosomal function and impair autophagy at the final step in the pathway [2]. Enthusiasm for repurposing HCQ as a clinical autophagy inhibitor was very high. Our team and a number of other groups initiated clinical trials to evaluate the safety and preliminary efficacy of HCQ-mediated autophagy inhibition as a strategy to increase the efficacy of other clinical anticancer agents and radiation therapy. To date, more than 30 early phase clinical trials with HCQ as a component of the regimen have been initiated and most are now completed. Unfortunately, these trials have yielded mostly underwhelming results [4, 5]. In the process of conducting these clinical trials, it became increasingly clear that HCQ could not inhibit autophagy potently enough in humans at doses that could be well tolerated to recapitulate the fantastic results that were seen in lab-based studies [2, 5]. New autophagy inhibitors with superior potency and more favorable safety profiles were urgently needed to objectively and comprehensively assess the true benefit of autophagy inhibition as a therapeutic approach.

There are many potentially successful strategies that can be used to obtain better inhibitors of known targets. Structure-based drug design, high-throughput screening of existing compounds, and innovative formulations are a few reasonable approaches. In our own efforts to develop improved autophagy inhibitors, we first made the decision to focus exclusively on lysosomal targeting. Although the autophagy cascade contains multiple attractive targets for pharmacological inhibition, some of them are known to be at least somewhat functionally redundant with each other. This could possibly lead to the undesired compensatory maintenance of autophagic degradation when a specific individual factor was targeted. However, all roads in autophagy eventually lead to the lysosome. Given its central role as a point of pathway convergence as well as its proven value as a therapeutic target in many HCQ-based preclinical studies, we felt it was an ideal choice for focused new agent development. We also knew that lysosomal targeting in terms of designing actual compounds was far from a shot in the dark. We had two FDA approved drugs that could be used as a basis for initial structure–activity relationship studies: HCQ and the anti-schistosomal drug lucanthone, which we previously showed caused lysosomal autophagy disruption [6].

Our medicinal chemistry efforts eventually yielded ROC-325, a dimeric small molecule that contains core motifs of HCQ and lucanthone (Fig. 1). Initial studies with ROC-325 showed that it had approximately tenfold greater anticancer activity and autophagic inhibition than HCQ against a broad range of tumor types with diverse genetic backgrounds [7]. Importantly, the structural features of ROC-325 elicited all of the hallmarks of autophagy inhibition while preserving the desired lysosomal disrupting properties that were characteristic of both HCQ and lucanthone.

**Fig. 1:**
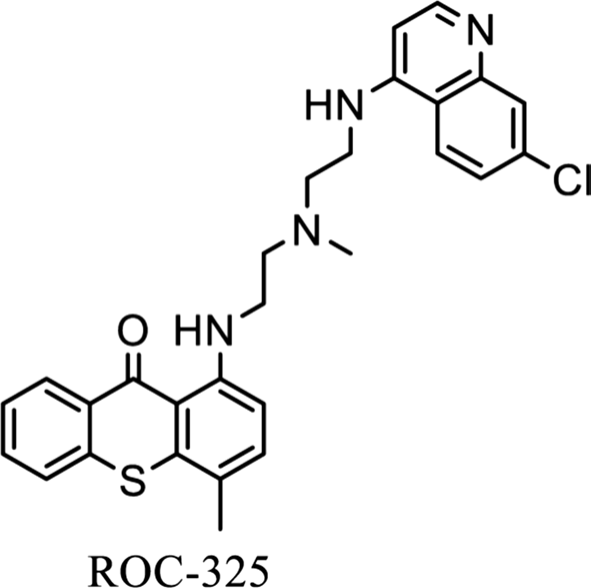
Chemical structure of ROC-325

We prioritized renal cell carcinoma (RCC) and acute myeloid leukemia (AML) for intensive investigations of the efficacy and pharmacodynamics of ROC-325 based on the high sensitivity of these cell types to ROC-325 in early screens as well as the relationship between mutations in the autophagy interactome and poor outcomes in both malignancies. In our first study, we confirmed the superior efficacy of ROC-325 over HCQ in RCC models and showed that ROC-325 could be administered orally on a daily schedule to mice bearing RCC xenografts for a prolonged period of time without inducing significant toxicity. In a subsequent study, we investigated the potential benefit of autophagy inhibition as a new approach to increase the anti-leukemic activity of the hypomethylating agent azacitidine (AZA) [8]. Recent studies have shown that autophagy plays a role in resistance to AZA, which is frequently used in the management of patients with myelodysplastic syndromes (MDS) and AML [9, 10]. We showed that the combination of ROC-325 and AZA blocked AZA-induced autophagy and yielded significantly improved activity against AML cell lines and primary AML blasts. Administration of the combination to leukemic mice was very well tolerated and resulted in significantly extended overall survival as compared to either monotherapy (*P* < 0.01) [8].

Autophagy inhibition remains a very attractive strategy to augment standard therapy and improve long-term outcomes for a broad range of malignancies. Our collective work, to date, has demonstrated that we are finally on a strategic trajectory to move beyond HCQ with respect to clinical autophagy inhibition. Our preclinical findings have established ROC-325 as a very promising novel lysosomal autophagy inhibitor. We are currently working to further refine its pharmacological properties with the goal of advancing our lead compound into a phase I clinical trial in the near future.

## Data Availability

Not applicable.

## Abbreviations

HCQ: hydroxychloroquine
FDA: US Food and Drug Administration
RCC: renal cell carcinoma
AML: acute myeloid leukemia
AZA: azacitidine
MDS: myelodysplastic syndromes
